# Requirements for translation re-initiation in *Escherichia coli*: roles of initiator tRNA and initiation factors IF2 and IF3

**DOI:** 10.1111/j.1365-2958.2008.06104.x

**Published:** 2008-03

**Authors:** Jae-Ho Yoo, Uttam L RajBhandary

**Affiliations:** Department of Biology, Massachusetts Institute of Technology Cambridge, MA 02139, USA

## Abstract

Despite its importance in post-transcriptional regulation of polycistronic operons in *Escherichia coli*, little is known about the mechanism of translation re-initiation, which occurs when the same ribosome used to translate an upstream open reading frame (ORF) also translates a downstream ORF. To investigate translation re-initiation in *Escherichia coli*, we constructed a di-cistronic reporter in which a firefly luciferase gene was linked to a chloramphenicol acetyltransferase gene using a segment of the translationally coupled *geneV–geneVII* intercistronic region from M13 phage. With this reporter and mutant initiator tRNAs, we show that two of the unique properties of *E. coli* initiator tRNA – formylation of the amino acid attached to the tRNA and binding of the tRNA to the ribosomal P-site – are as important for re-initiation as for *de novo* initiation. Overexpression of IF2 or increasing the affinity of mutant initiator tRNA for IF2 enhanced re-initiation efficiency, suggesting that IF2 is required for efficient re-initiation. In contrast, overexpression of IF3 led to a marked decrease in re-initiation efficiency, suggesting that a 30S ribosome and not a 70S ribosome is used for translation re-initiation. Strikingly, overexpression of IF3 also blocked *E. coli* from acting as a host for propagation of M13 phage.

## Introduction

Three pathways of translation initiation are known to operate in *Escherichia coli*: (i) *de novo* initiation, (ii) re-initiation and (iii) initiation with leaderless mRNAs. *De novo* initiation is the most frequent and best-understood pathway, occurring when a 30S ribosomal subunit binds to a mRNA containing a Shine–Dalgarno (SD) sequence, located 5–9 nucleotides upstream of the start codon of an open reading frame (ORF) (Gualerzi and Pon, 1990; Laursen *et al*., 2005). The second most frequent form of translation initiation is re-initiation. Re-initiation occurs when a ribosome that has completed translation of an upstream ORF in a polycistronic transcript remains bound to the mRNA and scans the mRNA in a bi-directional manner, as demonstrated in studies investigating translation of the overlapping lysis and coat protein genes in the RNA phage MS2 (Adhin and van Duin, 1990). The scanning ribosome can dissociate from the mRNA or re-initiate at a nearby start codon of a downstream ORF, positioned a few nucleotides away or overlapping with the stop codon from the preceding ORF (Adhin and van Duin, 1990). Re-initiation couples translation of a downstream gene to translation of an upstream gene, a phenomenon referred to as ‘translational coupling’ (Das and Yanofsky, 1984; Ivey-Hoyle and Steege, 1989; Spanjaard and van Duin, 1989; Adhin and van Duin, 1990). Translational coupling of a downstream gene can also occur due to unmasking of a SD sequence – normally inaccessible due to secondary structure – by ribosomes translating the upstream gene (de Smit and van Duin, 1993; Licis *et al*., 1998). Unmasking of the SD sequence allows ribosomes to independently bind and initiate translation of the downstream ORF [referred to as ‘facilitated binding’ (Rex *et al*., 1994)].

In eubacteria, many genes are part of polycistronic operons and appear to be coupled, as indicated by their proximity to each other. For example, greater than 25% of all operons in *E. coli* are polycistronic and ∼9% of the ORFs have a start codon overlapping with a stop codon from the preceding ORF (Blattner *et al*., 1997). While SD sequences are present in many of these downstream ORFs, their expression still appears to be tightly regulated by translational coupling (Govantes *et al*., 1998; Swain, 2004). Translational coupling and re-initiation are important for expression of proteins from polycistronic operons that code for proteins that are functionally related. Synthesis of multiprotein complexes such as ribosomes (Sor *et al*., 1987; Nomura, 1999), ATP synthetase (Rex *et al*., 1994), phages (Ivey-Hoyle and Steege, 1989) and photosynthetic complexes (Choudhary and Kaplan, 2000) require the co-ordinated expression of multiple proteins at specific ratios, so as to produce proteins in the amounts that are needed. Alterations in the normal stoichiometry can disrupt viral or cellular physiology, as demonstrated for replication of MS2 phage (Licis *et al*., 1998) and regulation of nitrogen fixation genes (Govantes *et al*., 1996).

*Escherichia coli* expresses three essential translation initiation factors – IF1, IF2 and IF3 – that are necessary for efficient and accurate *de novo* translation initiation. IF2 and IF3 are the best studied, and their specific roles in *de novo* translation initiation have been well characterized (Boelens and Gualerzi, 2002). Initiation factors, along with mRNA, initiator formylmethionyl-tRNA (fMet-tRNA^fMet^) and the 30S ribosomal subunit form the 30S initiation complex (IC), an intermediate required for *de novo* translation initiation. IF2 facilitates binding of the initiator fMet-tRNA^fMet^ to the P-site of the 30S IC (La Teana *et al*., 1996), while IF3 facilitates selection of the initiator tRNA and cognate initiation codon by destabilizing 30S ICs containing non-initiator tRNAs or non-canonical codon–anticodon pairing in the P-site (Hartz *et al*., 1990). Recent studies have also started to unravel the role of initiation factors in translation of leaderless mRNAs, which have zero or very few nucleotides upstream of the start codon. It has been suggested that translation initiation of leaderless mRNAs is mechanistically distinct from *de novo* initiation, specifically with regards to the role of initiation factors and the form of ribosome required (Moll *et al*., 2004). IF2 stabilizes 30S ICs containing leaderless mRNA, while IF3 destabilizes them (Grill *et al*., 2000; 2001). Elevated levels of IF3 also inhibit translation of leaderless mRNAs initiating with the canonical start codon AUG (Moll *et al*., 1998; Tedin *et al*., 1999). Several studies have also suggested that a 70S ribosome may be involved in translation initiation of leaderless mRNAs (Moll *et al*., 2004; Udagawa *et al*., 2004).

In spite of the prevalence of translation re-initiation, requirements in the initiator tRNA, initiation factors or the ribosome, for the assembly of ICs at re-initiation sites are not known. Although not required, SD sequences upstream of the re-initiation start codon enhance re-initiation efficiency (Das and Yanofsky, 1984; Spanjaard and van Duin, 1989; Ivey-Hoyle and Steege, 1992), while increasing the distance between the stop codon of the upstream ORF and the start codon of the downstream coupled ORF (intercistronic distance) decreases re-initiation efficiency (Ivey-Hoyle and Steege, 1989; Inokuchi *et al*., 2000; Karamyshev *et al*., 2004). There have been suggestions for the involvement of both 30S and 70S ribosomes in translation re-initiation (Martin and Webster, 1975; Petersen *et al*., 1978; Das and Yanofsky, 1984; Adhin and van Duin, 1990; Janosi *et al*., 1998; Inokuchi *et al*., 2000; Karamyshev *et al*., 2004; Moll *et al*., 2004), although there is no clear evidence for either hypothesis (for a review, see Janosi *et al*., 1996). The involvement of 70S ribosomes would exclude a role for IF3 in translation re-initiation (Spanjaard and van Duin, 1989; Moll *et al*., 2004), as IF3 is thought to only facilitate translation initiation from 30S ribosomal subunits. It has been shown, however, that certain mutations in IF3 enhanced translation initiation of a mutant *recJ* gene, which lacks a SD sequence and appears to be coupled to an upstream ORF by re-initiation (Haggerty and Lovett, 1997).

In this article, we have investigated the requirements for translation re-initiation in *E. coli*, by constructing a di-cistronic reporter based on the translationally coupled *geneV–geneVII* pair from M13 phage and studied the effects of using mutant initiator tRNAs or modulating IF2 and IF3 activity. We show that two of the unique properties of *E. coli* initiator tRNA – formylation of the amino acid attached to the tRNA and binding to the ribosomal P-site – are as important for re-initiation as for *de novo* initiation. Our results also show that IF2 is required for efficient re-initiation, whereas overexpression of IF3 decreased re-initiation efficiency and inhibited *E. coli* from acting as a host for M13 propagation. These results provide important insights into translation re-initiation in *E. coli.*

## Results

### Development of a tightly coupled di-cistronic reporter system

**Fig. 1:**
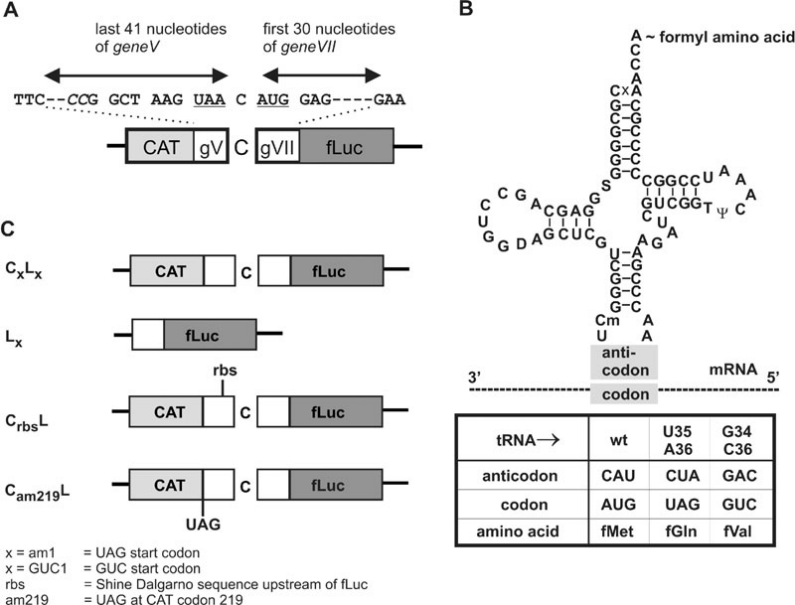
Coupled di-cistronic reporter system and initiator tRNAs. A. Schematic of the M13 *geneV–geneVII* (gV–gVII) intercistronic region fused in frame to chloramphenicol acetyltransferase (CAT) and firefly luciferase (fLuc) reporter genes, respectively, separated by a single C nucleotide. The stop and start codons for the CAT and fLuc reporters, respectively, are underlined. When required, the nucleotides in italics were changed to GA to create a Shine–Dalgarno sequence (GAGG) upstream of the fLuc gene. B. Structure of wild-type initiator tRNA_2_^fMet^ and anticodon mutants that decode UAG (U35A36 mutant) and GUC (G34C36 mutant) start codons with the resulting changes in aminoacylation. C. Schematic of wild-type and mutant, di-cistronic and mono-cistronic reporters. Capital letters refer to the specific reporter gene (C, CAT; L, Luciferase), while subscript acronyms refer to specific alterations in the reporters as indicated.

Expression of *geneVII*, encoding the structural coat protein for M13 phage, is tightly coupled to that of *geneV*, encoding an abundant single-stranded DNA-binding protein (Madison-Antenucci and Steege, 1998). The *geneVII* protein is expressed to lower levels compared with *geneV* and the translation initiation region upstream of *geneVII* has been described as an ‘inherently defective initiation site’, as it lacks a consensus SD sequence (Fig. 1A) and can only initiate by translational coupling (Ivey-Hoyle and Steege, 1992). We used the intercistronic region from *geneV–geneVII* to design and construct an inducible, di-cistronic reporter system to study translation re-initiation (Fig. S1). A 72-nucleotide-long sequence, encoding the last 13 amino acids of *geneV* and the first 10 amino acids of *geneVII*, was linked downstream of the chloramphenicol acetyltransferase (CAT) gene and upstream of the firefly luciferase (fLuc) gene respectively (Fig. 1A). These fusion reporters are, hereafter, referred to simply as CAT and fLuc respectively. The CAT and fLuc reporter genes are separated by a single C residue and represent a coupled di-cistronic operon, under transcriptional control of the inducible arabinose promoter (Fig. S1). The fLuc gene serves as a reporter for translation re-initiation, while the CAT gene allows us to monitor *de novo* initiation from the same transcript and normalize re-initiation activity to levels of ribosomes that enter the re-initiation site after translating the CAT gene.

### Mutant initiator tRNAs and mutant reporters

In addition to the di-cistronic reporter described above, we also used reporters in which the AUG initiation codons of the CAT and fLuc genes were mutated to UAG or GUC. Coexpression of wild-type (as a control) or anticodon sequence mutants of initiator tRNA_2_^fMet^ capable of decoding UAG (amber stop codon) or GUC (Val codon) as initiation codons was also necessary (Fig. 1B). The U35A36 (UAG decoding) and G34C36 (GUC decoding) mutant initiator tRNAs are aminoacylated by glutaminyl-tRNA synthetase (GlnRS) and valyl-tRNA synthetase (ValRS), to form Gln-tRNA^fMet^ and Val-tRNA^fMet^ respectively (Schulman and Pelka, 1985; Wu and RajBhandary, 1997). The aminoacyl-tRNAs are subsequently formylated by methionyl-tRNA formyltransferase (MTF) to formylglutaminyl-tRNA (fGln-tRNA^fMet^) and formylvalyl-tRNA (fVal-tRNA^fMet^) respectively (Fig. 1B). The mutant initiator tRNA genes were cloned into the reporter plasmids that contained the mutant reporter gene with the corresponding non-AUG start codon (Fig. 1C). Wild-type di-cistronic reporters are denoted as CL (letters corresponding to C for CAT and L for Luciferase), while mutant reporter genes are denoted by subscript acronyms after the letter corresponding to the reporter. Acronyms am1 and GUC1 refer to mutations of the AUG start codon to UAG and GUC respectively. The C_am219_L reporter refers to a mutant CAT reporter containing an internal amber codon at position 219 (Fig. 1C), separating the two reporter genes by 40 nucleotides. C_rbs_L denotes the creation of a SD or ribosome-binding sequence (rbs) in the C-terminal region of the CAT gene, 9 nucleotides upstream of the fLuc start codon (Fig. 1A and C). When necessary, additional expression vectors containing genes for initiation factors (IF2 and IF3), methionyl-tRNA synthetase (MetRS) or MTF were co-transformed with the di-cistronic reporter.

### Characterization of the coupled di-cistronic reporter system

**Fig. 2:**
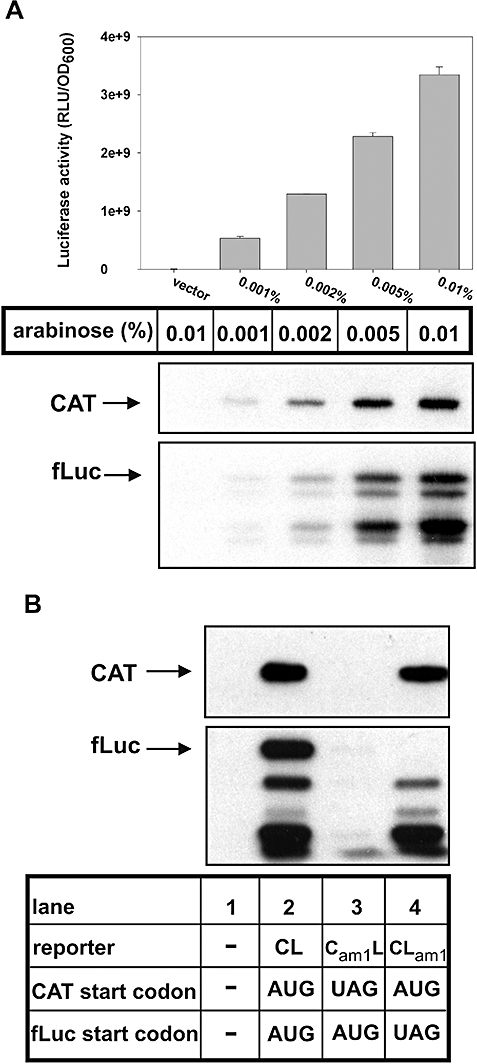
Characterization of di-cistronic reporter. A. (Top) fLuc activity in extracts of *E. coli* CA274 cells transformed with the wild-type di-cistronic reporter (CL) or the empty vector (vector), induced with increasing concentrations of arabinose. fLuc activity is reported as relative luminescence units (RLU)/OD_600_. (Bottom) Immunoblots of cell extracts with anti-CAT or anti-fLuc antibody (Ab). B. Immunoblots with anti-CAT or anti-fLuc Ab of *E. coli* CA274 cell extracts containing empty vector, CL, C_am1_L or CL_am1_.

**Fig. 3:**
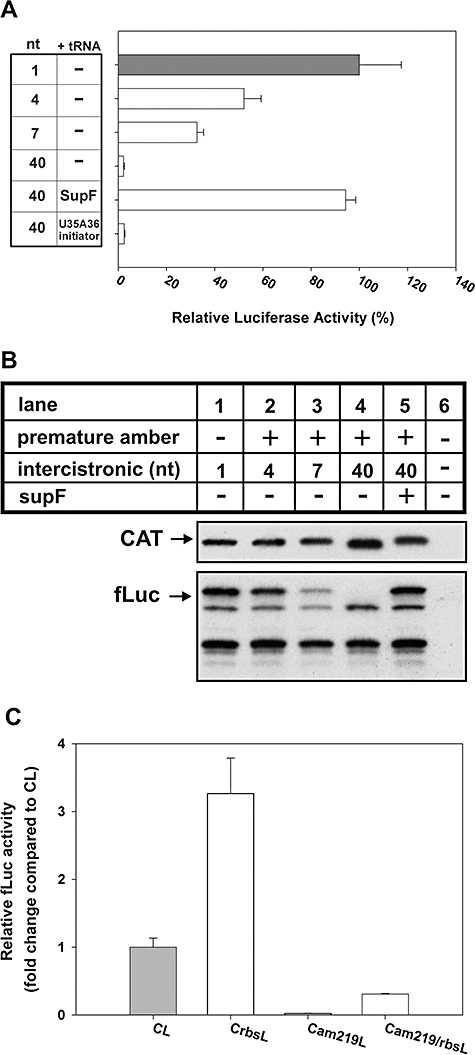
The fLuc reporter gene is translated by re-initiating ribosomes. A. Relative fLuc activity from mutant di-cistronic reporters containing premature UAG stop codons placed at various distances upstream of the normal *geneV* UAA stop codon. SupF suppressor or U35A36 mutant initiator tRNA was also coexpressed. fLuc activity from CL was set at 100%. nt, number of nucleotides separating the two reporter genes. fLuc activity is defined as RLU/OD_600_. B. Immunoblot of total-cell extract from (A) using anti-CAT or anti-fLuc Ab. The mutant di-cistronic reporter containing a 40-nucleotide intercistronic region (C_am219_L, lane 4) encodes a CAT protein that is ∼1.4 kDa smaller than the full-length CAT. Lane 6 represents a sample from *E. coli* transformed with empty vector. C. Relative fLuc activity from mutant di-cistronic reporters without or with a SD sequence upstream of the fLuc start codon (C_rbs_L). fLuc activity from CL was set at 1. fLuc activity is defined as RLU/OD_600_.

The di-cistronic reporter system was characterized to confirm that both reporters, CAT and fLuc, were co-transcribed and co-translated. *E. coli* CA274 cells were transformed with the wild-type di-cistronic reporter CL, induced with arabinose, and cell extracts analysed for CAT and fLuc activity, and for protein expression levels using immunoblot analysis. Assays for fLuc activity showed a parallel increase in activity with increasing levels of arabinose (Fig. 2A, graph). Immunoblot analyses revealed that expression of both reporters increased in a similar manner with increasing levels of arabinose (Fig. 2A, immunoblot), consistent with both genes being co-transcribed and co-translated. Several faster migrating bands representing internally initiated luciferase fragments were also detected with the anti-fLuc antibody (Ab). These bands do not represent degradation products derived from full-length fLuc and contribute little, if any, to fLuc activity (compare levels of truncated fragments in Fig. 3B, lane 4, with their activity in Fig. 3A).

To verify that fLuc expression was coupled to CAT expression, a series of experiments were performed. We constructed mutant reporters with the start codon for either reporter altered to UAG (C_am1_L and CL_am1_). *E. coli* CA274 cells were transformed with these plasmids and cell extracts analysed for protein expression using immunoblot analysis (Fig. 2B). With C_am1_L, in which the start codon of the CAT gene was altered to UAG, there was no detectable CAT expression as expected, but fLuc expression was also abolished (Fig. 2B, lane 3), consistent with translational coupling of fLuc to translation of CAT. It is also possible, however, that the absence of fLuc expression from C_am1_L is due to reduced levels of mRNA as a result of transcriptional polarity (Nudler, 2002) and/or accelerated mRNA degradation (Iost and Dreyfus, 1995). In contrast, with CL_am1_, in which the start codon for fLuc was mutated to UAG, expression of full-length fLuc was abolished, while expression of CAT and internally initiated luciferase fragments were unaffected (Fig. 2B, lane 4). These data show that the fLuc reporter is translated from the start codon originally derived from *geneVII* (Fig. 1A).

For a more direct demonstration of translation re-initiation of the fLuc gene, we increased the intercistronic distance between the stop codon of the CAT gene and the start codon of the fLuc gene. Coupling efficiency – a measure of the fraction of ribosomes that terminate translation and then re-initiate – generally decreases with increasing intercistronic length, likely due to increased probability of ribosomes dissociating from the mRNA before arriving at an appropriate re-initiation site (Das and Yanofsky, 1984; Spanjaard and van Duin, 1989; Adhin and van Duin, 1990). A downstream gene within a polycistronic operon that is translated independently is, on the other hand, largely unaffected by increases in intercistronic distance (Ivey-Hoyle and Steege, 1989; Madison-Antenucci and Steege, 1998). We constructed additional mutant reporters, each containing a premature UAG stop codon at different positions within the CAT gene, to increase the intercistronic distance between the CAT and fLuc reporter genes from 1 nucleotide to 4, 7 and 40 nucleotides (Fig. 3A). fLuc activity decreased with increasing intercistronic distance, with a 40-nucleotide separation in the C_am219_L reporter decreasing activity down to background levels (< 3% activity, Fig. 3A). Co-expression of SupF, an amber suppressor tRNA, restored translational coupling and fLuc activity from the C_am219_L reporter (Fig. 3A). A 4-nucleotide separation caused a twofold decrease in fLuc activity, a level of reduction similar to previously reported effects on the native *geneV–geneVII* pair from the related f1 phage (Ivey-Hoyle and Steege, 1989). Cell extracts prepared from samples in Fig. 3A were also analysed by immunoblotting (Fig. 3B). Consistent with fLuc activity, immunoblots revealed decreasing levels of full-length fLuc protein with increasing intercistronic distance (Fig. 3B, lanes 1–4). Meanwhile, levels of CAT protein and internally initiated fLuc protein fragments remained essentially unchanged; therefore decreased expression of full-length fLuc is not due to changes in mRNA levels.

Shine–Dalgarno sequences upstream of translationally coupled genes have been shown to increase translation re-initiation efficiency (Das and Yanofsky, 1984; Spanjaard and van Duin, 1989). To determine if a SD sequence would increase translation re-initiation of the fLuc reporter, we created a 4-base pair SD sequence (GAGG), 9 nucleotides upstream of the fLuc start codon (C_rbs_L, Fig. 1A and C). The addition of the SD sequence increased fLuc activity greater than threefold (Fig. 3C). Immunoblot analysis showed that the increase in fLuc activity was due to an increase in re-initiation efficiency as levels of CAT protein from C_rbs_L was unaffected by the creation of the SD sequence (data not shown). To determine if the newly created SD sequence specifically increased re-initiation, as opposed to increased *de novo* or independent initiation of the fLuc gene (i.e. binding of free ribosomes to the new SD sequence), the mutant reporter C_am219/rbs_L was constructed by introducing a SD sequence into the intercistronic region of the uncoupled di-cistronic reporter C_am219_L. Any increase in fLuc expression from C_am219/rbs_L due to creation of a SD sequence should reflect *de novo* initiation activity. Creation of a SD sequence in C_am219/rbs_L did increase fLuc activity, relative to the uncoupled reporter C_am219_L (Fig. 3C). However, the relative fLuc activity from C_am219/rbs_L was less than 10% of the activity obtained from the coupled reporter containing a SD sequence (C_rbs_L). Thus, introduction of an intercistronic SD sequence mostly increased the re-initiation efficiency.

In summary, the above results indicate that translation of the fLuc reporter almost exclusively utilizes re-initiating ribosomes for initiation, even in the presence of a SD sequence. The results also show that the synthetic di-cistronic operon retains many of the properties of the native *geneV–geneVII* pair from M13 phage and allows for specific analysis of *de novo* initiation and re-initiation.

### Activity of mutant initiator tRNAs in re-initiation

The specific requirements of translation re-initiation in an initiator tRNA or for initiation factors are unknown. The development of a coupled reporter system to specifically monitor re-initiation allowed us to study the effects of utilizing mutant initiator tRNAs or modulating the activity of initiation factors to evaluate their roles in re-initiation *in vivo*.

**Fig. 4:**
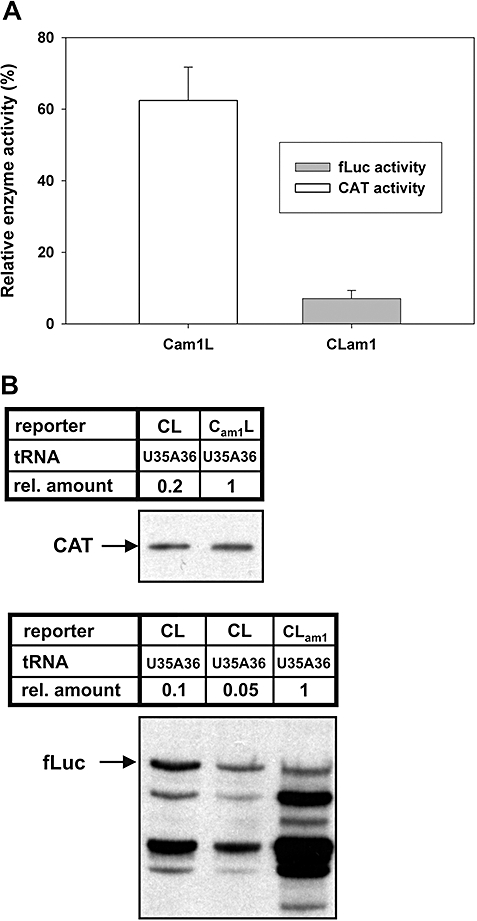
*De novo* and re-initiation of di-cistronic reporters using UAG as the initiation codon. A. Relative enzyme activity from *E. coli* CA274 transformed with CL or mutant di-cistronic reporters containing either a mutant CAT reporter (C_am1_L) or a mutant fLuc reporter (CL_am1_) and expressing the U35A36 mutant initiator tRNA. Cell extracts were analysed for CAT (C_am1_L, white bar) or fLuc activity (CL_am1_, grey bar) and compared with activity from CL. CAT activity was determined as described in *Experimental procedures* and normalized to total protein and β-lactamase activity (to normalize for plasmid copy number). fLuc activity was determined as described in *Experimental procedures* and normalized to cell number and CAT activity. B. Immunoblots of cell extracts from (A), using anti-CAT (top) or anti-fLuc (bottom) Ab. Different relative amounts of cell extract were analysed to facilitate comparison of protein levels between wild-type and mutant samples.

Changing the CAU anticodon of wild-type initiator tRNA to CUA (U35A36 mutant) or GAC (G34C36 mutant) allows the mutant initiator tRNA to initiate from UAG and GUC codons, respectively, in *E. coli* (Fig. 1B) (Varshney and RajBhandary, 1990; Wu and RajBhandary, 1997). The corresponding G34C36 mutant initiator tRNA can, similarly, be used to initiate *de novo* protein synthesis from GUC codons in a mutant CAT gene in mammalian cells (Drabkin and RajBhandary, 1998) and in a mutant leaderless bacterio-opsin gene in the archaeon *Halobacterium salinarum* (Srinivasan *et al*., 2006). Prior to testing if UAG could also be used as a start codon for translation re-initiation, we confirmed that the mono-cistronic reporter L_am1_ could be initiated from UAG by *de novo* initiation (∼100% efficiency, see Fig. S2). We proceeded to compare the efficiency of the U35A36 mutant initiator tRNA in *de novo* initiation of mutant CAT from C_am1_L and re-initiation of mutant fLuc from CL_am1_. Based on enzyme activity, the mutant CAT reporter was translated to ∼60% efficiency, while the mutant fLuc reporter was translated to ∼7% efficiency (Fig. 4A) (the efficiencies were calculated relative to enzyme activity from the wild-type reporter, CL). We noticed, however, that there was a discrepancy between mutant CAT enzyme activity and protein levels. Immunoblot analysis showed an almost fivefold reduction in CAT protein levels in cells expressing the C_am1_L reporter (Fig. 4B, top) compared with those expressing the wild-type CL reporter, instead of the ∼1.6-fold reduction expected on the basis of CAT activities (Fig. 4A). These results suggest that the specific activity of the mutant CAT protein initiated with formylglutamine and extended at the C-terminus by 13 amino acids, is higher by a factor of ∼3 than wild-type CAT protein initiated with formylmethionine, leading to an overestimation of CAT expression based on enzyme activity. To correct for this inconsistency, we used immunoblotting and densitometric analysis of the immunoblots to calculate the translation initiation efficiencies for mutant CAT expression (Table S1). Use of this assay revealed that the U35A36 mutant initiator tRNA was still more efficient in *de novo* initiation of mutant CAT (17%) than in re-initiation of the mutant fLuc reporter (7%). The specific activity of the fLuc reporter was not affected by the type of amino acid present at the N-terminus (compare Fig. 4A and B, bottom). Interestingly, a non-fusion, native CAT reporter previously used in our laboratory did not exhibit differences in specific activity when initiating with amino acids other than methionine (Varshney and RajBhandary, 1990; Mangroo and RajBhandary, 1995; Mayer *et al*., 2003). This was also verified experimentally during this work (data not shown).

It is unclear why the overall efficiency of the U35A36 mutant initiator tRNA was lower in *de novo* translation of CAT and re-initiation of fLuc from the di-cistronic reporter system (17% and 7% respectively) compared with efficiencies previously observed with a *de novo* initiated mono-cistronic native CAT reporter (60–80%) (Varshney and RajBhandary, 1990) or the mono-cistronic mutant fLuc reporter (100%, Fig. S2). We attribute this difference to reporter-specific effects of mono-cistronic versus di-cistronic systems and differences in activity of the U35A36 mutant initiator tRNA in *de novo* initiation versus re-initiation. Notwithstanding this difference, the results obtained showed that non-canonical start codons could be used for studying translation re-initiation.

### Requirements in initiator tRNA for translation re-initiation

Eubacterial initiator tRNAs have several unique properties that distinguish them from elongator tRNAs. Two of these properties (i) formylation of Met-tRNA^fMet^ to fMet-tRNA^fMet^ by MTF and (ii) binding of fMet-tRNA^fMet^ to the ribosomal P-site are crucial for activity of the initiator tRNA in initiation. The key elements in the initiator tRNA important for specifying these distinctive properties have been identified. These include a mismatch at the end of the acceptor stem for recognition by MTF and three consecutive G:C base pairs in the anticodon stem for binding to the ribosomal P-site (RajBhandary, 1994) (Fig. S3A).

The availability of well-characterized mutant initiator tRNAs and the finding above that UAG could be used as a codon for translation re-initiation *in vivo* enabled us to ask whether the requirements in an initiator tRNA for translation re-initiation are the same as for *de novo* initiation. More specifically, how important is formylation of the initiator tRNA for re-initiation, and how important is the ability of the initiator tRNA to bind to the ribosomal P-site for re-initiation? The mutant initiator tRNAs used were the U35A36/G72G73 (G72G73) mutant defective in formylation (Varshney *et al*., 1991a), and the C30:G40/U35A36 (C30G40) and the U29C30A31:U39G40A41/U35A36 (3GC) mutants (Seong and RajBhandary, 1987; Mangroo and RajBhandary, 1995) (Fig. S3A) defective in binding of the tRNA to the ribosomal P-site (Mandal *et al*., 1996). Acid urea polyacrylamide gel electrophoresis (Varshney *et al*., 1991a), followed by Northern blot analysis of the mutant tRNAs isolated from cells, confirmed that the G72G73 mutant initiator tRNA is completely aminoacylated but not formylated, whereas the C30G40 and 3GC mutant initiator tRNAs were completely aminoacylated and formylated (Fig. S3B).

**Fig. 5:**
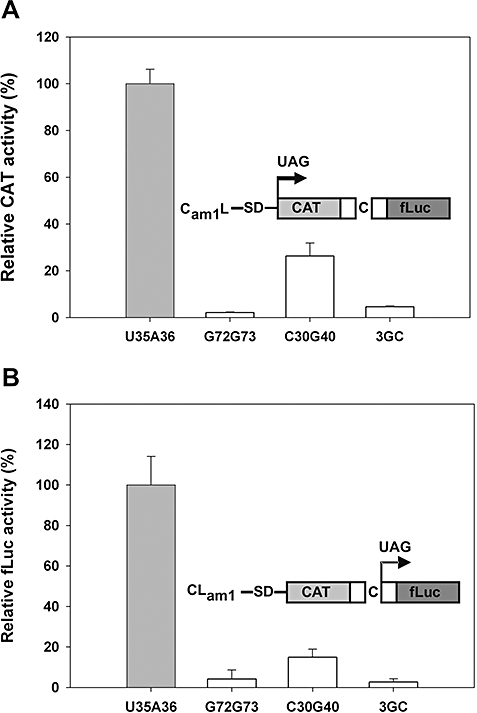
*De novo* and re-initiation efficiency of mutant initiator tRNAs. A. Relative CAT activity from *E. coli* CA274 transformed with the C_am1_L reporter, expressing a mutant CAT gene initiating with a UAG codon and the U35A36 mutant initiator tRNA (grey bar) or the U35A36 mutant tRNA containing additional mutations as noted (white bars). Activity from the U35A36 mutant initiator tRNA was set at 100% and CAT activity defined as in legend to Fig. 4A. B. Relative fLuc activity from *E. coli* CA274 transformed with the CL_am1_ reporter, expressing a mutant fLuc gene initiating with a UAG codon, and the U35A36 mutant initiator tRNA (grey bar) or the U35A36 mutant initiator tRNA containing additional mutations as noted (white bars). Activity from the U35A36 mutant initiator tRNA was set at 100% and fLuc activity is as defined in legend to Fig. 4A.

*Escherichia coli* CA274 were transformed with either the C_am1_L or the CL_am1_ reporter carrying one or the other of the mutant initiator tRNA genes, and extracts assayed for CAT and fLuc activity levels. As shown previously, the G72G73 and 3GC mutant initiator tRNAs were much less active in *de novo* synthesis of CAT, while the activity of the C30G40 mutant initiator tRNA was slightly higher but still significantly lower than that of the original U35A36 mutant initiator tRNA (Fig. 5A) (Varshney *et al*., 1991b; Mandal *et al*., 1996). The activities of the mutant tRNAs in translation re-initiation were essentially the same as in *de novo* initiation (compare Fig. 5A and B) in terms of both the requirements for formylation of the tRNA (the G72G73 mutant) and direct binding of the tRNA to the ribosomal P-site (the C30G40 and the 3GC mutants). As shown below, these results, although indirect, also imply important roles for IF2 and IF3 in re-initiation.

### IF2 activity is important for efficient re-initiation

**Fig. 6:**
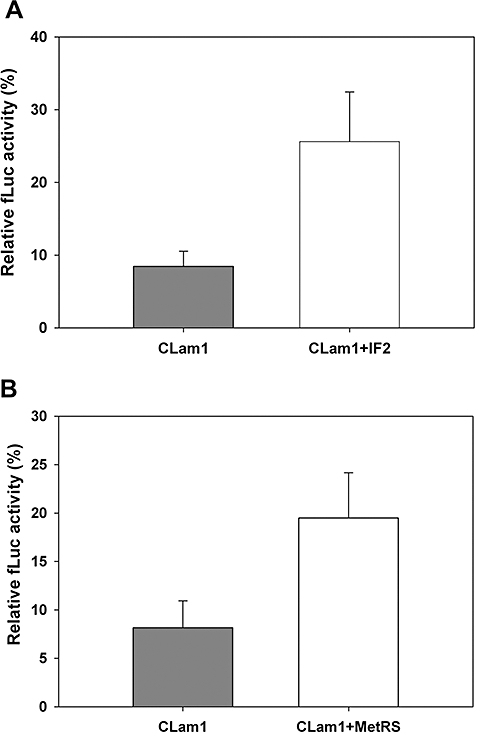
Overexpression of IF2 and MetRS enhances translation re-initiation with the U35A36 mutant initiator tRNA. A. Relative fLuc activity from *E. coli* CA274 co-transformed with the mutant di-cistronic reporter (CL_am1_) and a compatible expression plasmid without (grey bar) or with IF2 gene (white bar). fLuc activity from the CL reporter co-transformed with or without the IF2 expression plasmid was set at 100%, with all reporters expressing U35A36 mutant initiator tRNA. fLuc activity is defined as in the legend to Fig. 4A. B. Same as (A), except cells were co-transformed with a plasmid expressing MetRS (white bar).

Binding of IF2 to initiator tRNA is influenced not only by the formyl group (Sundari *et al*., 1976), but also by the identity of the amino acid attached to the tRNA (Mayer *et al*., 2003). Of the amino acids tested, IF2 showed the highest affinity for mutant initiator tRNA carrying formylmethionine (fMet) or formylvaline (fVal) and the lowest for formylglutamine (fGln) (Wu and RajBhandary, 1997; Mayer *et al*., 2003). The importance of IF2 for efficient *de novo* initiation was demonstrated by the increased initiation activity observed with the U35A36 mutant initiator tRNA (aminoacylated with fGln) in cells overproducing IF2 (Mangroo and RajBhandary, 1995; Mayer *et al*., 2003). In contrast, overproduction of IF2 had minimal effects on *de novo* initiation activity with the G34C36 mutant initiator tRNA carrying fVal. We tested the effects of overproducing IF2 on re-initiation of the mutant fLuc reporter using the U35A36 mutant initiator tRNA. As shown in Fig. 6A, overproduction of IF2 increased the re-initiation efficiency of the U35A36 mutant initiator tRNA about 2.5-fold.

Overexpression of MetRS leads to aminoacylation of the U35A36 mutant initiator tRNA with methionine instead of glutamine (Varshney and RajBhandary, 1992) and thereby increases its affinity for IF2 (Wu and RajBhandary, 1997). As shown in Fig. 6B, overexpression of MetRS also resulted in increased synthesis of mutant fLuc with the U35A36 mutant initiator tRNA, confirming the importance of IF2 in efficient translation re-initiation.

**Fig. 7:**
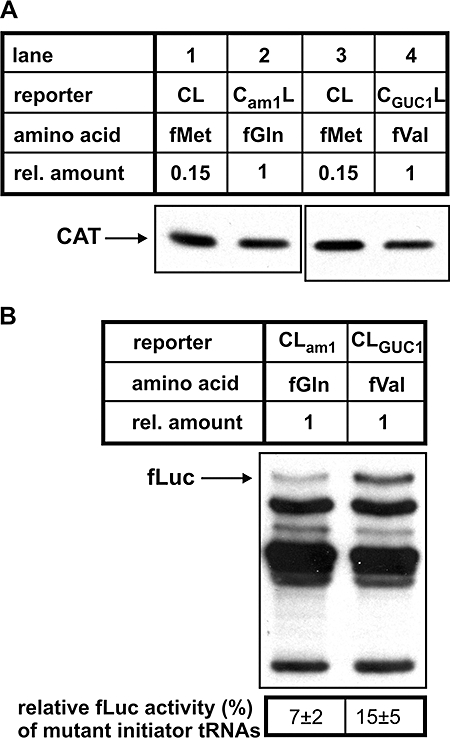
Mutant initiator tRNA with higher affinity for IF2 is more active in re-initiation. A. Immunoblot with anti-CAT Ab of *E. coli* CA274 cells transformed with CL, C_am1_L or C_GUC1_L and coexpressing the corresponding mutant initiator tRNAs. B. Immunoblot with anti-fLuc Ab of *E. coli* CA274 cells containing the CL_am1_ and CL_GUC1_ reporters and coexpressing the corresponding mutant initiator tRNA. fLuc activity assays were also performed and reported as percentage activity, relative to the CL reporter set at 100%. fLuc activity is as defined in legend to Fig. 4A.

Unlike the U35A36 mutant initiator tRNA, the activity of the G34C36 mutant initiator tRNA, aminoacylated with fVal, is not limited by its affinity for IF2 (Wu *et al*., 1996). To further investigate the role of IF2 in re-initiation, we compared the activities of the U35A36 and G34C36 mutant initiator tRNAs in *de novo* initiation and re-initiation of mutant reporters initiating with UAG or GUC codons. Both mutant initiator tRNAs displayed comparable *de novo* translation activity in synthesizing mutant CAT (Fig. 7A, compare lanes 1 and 2 with lanes 3 and 4). However, when we compared the efficiencies of both mutant initiator tRNAs in re-initiation of mutant fLuc reporters, the data showed that the G34C36 mutant initiator tRNA was more efficient in translation re-initiation (Fig. 7B). Overall, our results show that IF2 is required for efficient re-initiation *in vivo* and also suggest that re-initiation, at least with our system, may have a greater requirement for IF2 than *de novo* initiation.

### Overexpression of IF3 decreases efficiency of re-initiation

IF3 performs multiple functions during *de novo* initiation to ensure accurate translation, in addition to keeping ribosomal subunits separated through its anti-association activity (Boelens and Gualerzi, 2002). Elevated levels of IF3 *in vivo* cause minimal effects on *de novo* initiation from canonical start codons but inhibit translation from non-canonical start codons (Sacerdot *et al*., 1996; O'Connor *et al*., 2001; Petrelli *et al*., 2001).

**Fig. 8:**
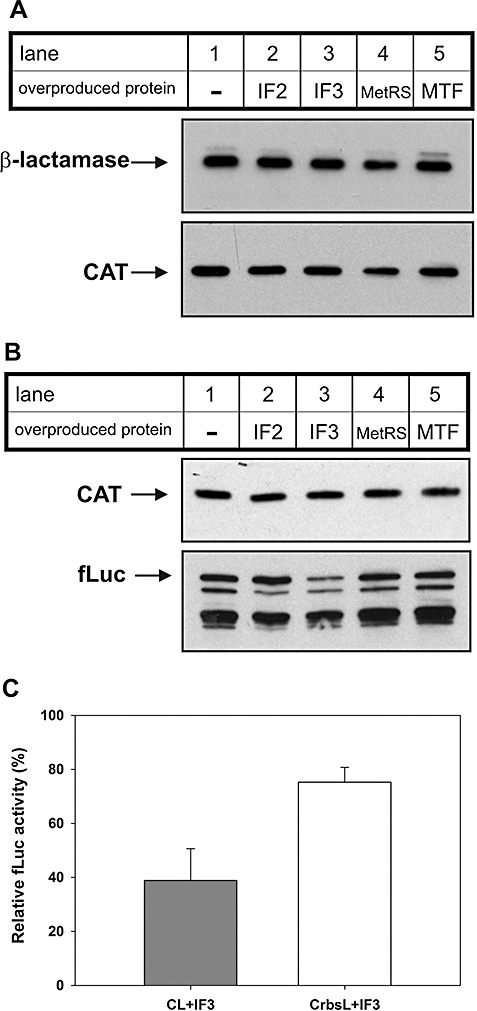
Overexpression of IF3 reduces re-initiation efficiency. A and B. *E. coli* CA274 cells were co-transformed with CL and expression plasmids that either were empty (−), or contained genes encoding IF2, IF3, MetRS or MTF. Cell extracts were analysed using immunoblots with anti-β-lactamase or anti-CAT Ab (A), or anti-CAT and anti-fLuc Ab (B). C. *E. coli* CA274 cells were co-transformed with CL or C_rbs_L, and a compatible expression plasmid that either was empty or contained the IF3 gene. fLuc activity is presented as percentage change in fLuc activity in cells overexpressing IF3, relative to cells not overexpressing IF3. fLuc activity is as defined in legend to Fig. 4A.

We tested the effects of overexpressing IF3, as well as other proteins of the translational machinery including IF2, MetRS and MTF, on translation of CAT and fLuc from the CL reporter. *De novo* translation of CAT was essentially unaffected by the overproduction of any of the translation factors (Fig. 8A, also Fig. S4, top). In contrast, we observed an almost threefold decrease in fLuc activity in cells overproducing IF3, and consistent with our findings above, a slight increase in fLuc activity in cells overproducing IF2 (Fig. 8B and Fig. S4, bottom). As fLuc activity was normalized to CAT activity, these differences reflected changes in re-initiation efficiency. The results suggested that high levels of IF3 either prevented two out of three ribosomes that would normally re-initiate at the fLuc AUG start codon from doing so or reduced the rate of re-initiation by a factor of 3. Control experiments also showed that *de novo* initiation of fLuc from the mono-cistronic L reporter was unaffected by overexpression of IF3 (Fig. S5).

Excess IF3 is also known to inhibit translation of leaderless mRNAs, even those with canonical start codons (Grill *et al*., 2001). One explanation proposed for this effect is that IF3 destabilizes 30S ICs containing leaderless mRNAs due to the lack of a SD sequence (Moll *et al*., 1998; Tedin *et al*., 1999; Boelens and Gualerzi, 2002). We therefore investigated whether a SD sequence upstream of the coupled fLuc gene would impact IF3-mediated inhibition of translation re-initiation. The inhibitory effects of overexpression of IF3 on re-initiation were less severe when a SD sequence was present (Fig. 8C). Thus, IF3 may have a similar role in regulating translation initiation from leaderless mRNAs and translation re-initiation in the absence of a SD sequence.

### Overexpression of IF3 impairs propagation of M13 phage

**Fig. 9:**
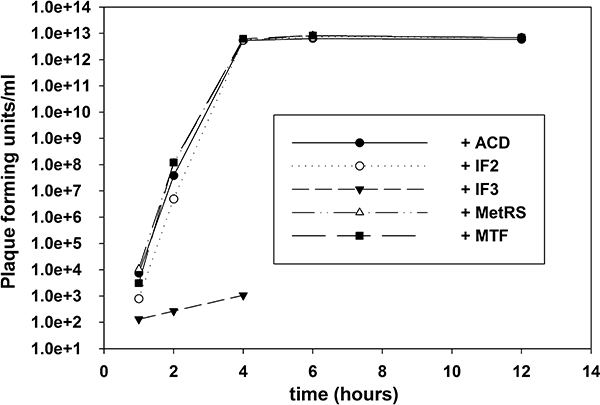
Overexpression of IF3 interferes with propagation of M13 phage. Phage titres from *E. coli* CA274 transformed with empty vector (ACD), or vector containing genes for IF2, IF3, MetRS or MTF and infected with M13 in liquid culture for the times indicated. Phage titres were determined from the supernatants of the infected cultures.

As shown above, overproduction of IF3 altered the efficiency of translation re-initiation from the di-cistronic reporter, whose intercistronic region was originally derived from a M13 operon. This result raised the question of whether overproduction of IF3 could also reduce the levels of *geneVII* protein made in cells infected with M13 phage. We therefore investigated the effect of overproduction of IF3 on propagation of M13 phage in *E. coli*. We infected *E. coli* CA274 cells overproducing IF3 or other proteins involved in protein translation with M13 phage. Interestingly, *E. coli* overproducing IF3 was severely compromised in their ability to act as hosts for propagation of M13 phage as indicated by the very low phage titres obtained (Fig. 9). In contrast, *E. coli* CA274 cells transformed with plasmids overproducing IF2, MetRS or MTF were not significantly affected in their ability to host phage propagation (Fig. 9).

Overexpression of IF3 did not significantly affect the growth rate of *E. coli* when compared with any of the other control transformants (data not shown). To account for the extremely slow propagation of M13 in *E. coli* overexpressing IF3, we investigated whether the step affected was phage adsorption or replication and/or assembly of phage inside the cell. The results obtained suggest that overproduction of IF3 interferes with a step involved in phage replication and/or assembly inside the cell but not phage adsorption to the cell (data not shown).

## Discussion

### A coupled di-cistronic reporter system for studying translation re-initiation

**Fig. 10:**
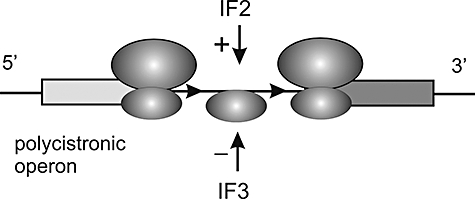
Model for translation re-initiation in *E. coli.* Proposed molecular events occurring between translation termination at the upstream gene (light grey bar) and subsequent re-initiation at the downstream gene (dark grey bar). IF2 enhances re-initiation efficiency, while elevated levels of IF3 decrease it. Our results also suggest that a 30S ribosomal subunit is used for accurate translation re-initiation.

We have shown that the di-cistronic CAT-fLuc reporter developed here is an excellent system for studying translation re-initiation. Using anticodon mutants of initiator tRNA (Fig. 1B), we demonstrate that a mutant fLuc reporter gene can be translated by re-initiation from non-AUG initiation codons (Figs 4 and 7). Introduction of additional mutations elsewhere in the initiator tRNA allowed us to identify components of the translation initiation machinery involved in re-initiation. The importance of formylation of the initiator tRNA for its activity in re-initiation indicates that IF2 is required for re-initiation, while a requirement for three consecutive G:C base pairs in the anticodon stem of the initiator tRNA indicates that IF3 also plays a role in re-initiation. Re-initiation appears to have a higher requirement for IF2 than *de novo* initiation, whereas overproduction of IF3 inhibits re-initiation. Thus, as suggested for leaderless mRNAs, the relative levels of IF2 and IF3 may also influence translation re-initiation efficiency (Fig. 10).

### Role of IF2 and SD sequence in translation re-initiation

The effect of overproduction of IF2 on activity of the U35A36 mutant initiator tRNA in re-initiation shows that re-initiation is influenced by the intrinsic affinity of initiator tRNA for IF2 (Fig. 6). Decreases in affinity of the initiator tRNA for IF2 (such as aminoacylation of the initiator tRNA with glutamine) are likely to reduce the probability of an initiator tRNA being properly positioned in the P-site to base pair with the start codon and initiate 30S IC formation, before the re-initiating ribosome drops off the mRNA or continues scanning.

Introduction of a SD sequence upstream of the fLuc gene increased re-initiation efficiency (Fig. 3C), presumably by anchoring the ribosome so that it can position a start codon in the P-site (Gualerzi and Pon, 1990). Alternatively, SD sequences may stabilize 30S ICs after codon–anticodon pairing has already been established (Studer and Joseph, 2006). Either mechanism would explain the low efficiency of translation re-initiation in the absence of a SD sequence, despite the fact that re-initiating ribosomes are already bound to the mRNA.

### Role of IF3 in translation re-initiation

Overexpression of IF3 inhibits translation initiation from leaderless mRNA (Tedin *et al*., 1999). Two different hypotheses have been put forth to explain this inhibition. One hypothesis suggests that the anti-subunit association activity of IF3 reduces the levels of free 70S ribosomes implicated in translation of leaderless mRNAs (Udagawa *et al*., 2004). An alternative hypothesis suggests that IF3 binds to the 30S subunit and destabilizes all 30S ICs containing leaderless mRNAs (even mRNAs containing an AUG start codon), due to the absence of SD–anti-SD and/or S1 ribosomal protein-mediated interactions between the ribosome and leaderless mRNA (Moll *et al*., 1998; Tedin *et al*., 1999).

Our finding that elevated levels of IF3 inhibit translation re-initiation of the fLuc reporter starting with an AUG codon (Fig. 8B) is unlikely to be due to reduced levels of free 70S ribosomes, as re-initiating ribosomes do not originate from free pools (Fig. 3). A more plausible explanation is that the re-initiating 30S ribosome·mRNA·fMet-tRNA complex is weak for lack of a SD sequence and IF3 inhibits re-initiation by destabilizing this complex and/or decreasing the rate of 50S subunit association (Antoun *et al*., 2006).

These are three instances where elevated levels of IF3 inhibit translation initiation in *E. coli*: (i) *de novo* initiation involving non-canonical start codons and/or non-initiator tRNAs, (ii) translation of leaderless mRNAs and (iii) re-initiation in the absence of an upstream SD sequence. In the latter two cases, where initiation involves an AUG codon and initiator tRNA^fMet^, but no SD sequence, IF3 may be acting as a fidelity factor to minimize unintended or spurious initiation events (O'Connor *et al*., 2001). Otherwise, the mere presence of a canonical start codon (AUG, GUG, UUG) near the 5′ end of any mRNA or proximal to the stop codon of an ORF could lead to translation of random unintended ORFs.

In addition to IF3's role in discriminating between canonical and non-canonical initiation codons, it is known that changes in IF3 activity cause pleiotrophic effects, possibly due to alterations in the stoichiometry of gene products synthesized from polycistronic operons. Specific mutations in the IF3 gene of *Rhodobacter sphaeroides* and *Myxococcus* x*anthus* result in reduced synthesis of the photosynthetic complex and impaired differentiation and sporulation of *M. xanthus* respectively (Cheng *et al*., 1994; Babic *et al*., 1997). Many of the proteins required for the formation of *R. sphaeroides* photosynthetic complexes or for differentiation/sporulation of *M. xanthus* are expressed from polycistronic mRNAs (Choudhary and Kaplan, 2000; Viswanathan *et al*., 2007). Regarding the striking effects of IF3 overproduction on M13 phage propagation (Fig. 9), decreased expression of *geneVII* alone could cause significant delays in phage production resulting in extremely low phage titres. Expression of some other M13 phage gene(s) may also be sensitive to IF3 levels or overproduction of IF3 could affect the synthesis of one or more host proteins necessary for M13 propagation.

### Form of ribosome used for re-initiation *in vivo*

Another important question is whether the assembly of a 70S IC at the initiation codon for translation re-initiation begins with a 30S or a 70S ribosome (Janosi *et al*., 1996). Re-initiation with a 70S ribosome would mean that IF3 plays no role in recognition of the initiator tRNA (Spanjaard and van Duin, 1989; Moll *et al*., 2004; Lancaster and Noller, 2005). However, as described above, we and others (Haggerty and Lovett, 1997) have shown that IF3 is involved in translation re-initiation (Fig. 5B). Sensitivity of translation re-initiation to elevated levels of IF3 suggests that a 30S ribosome subunit is used for translation re-initiation *in vivo* (Fig. 8B). One of the roles of IF3 in translation initiation is to facilitate the binding of initiator tRNA to the ribosomal P-site. Binding of IF3 to the 30S ribosome is thought to bring about a conformational change of the 16S rRNA and position A790 of helix h24, and G1338 and A1339, for interaction with the three conserved G:C base pairs in the anticodon stem of the initiator tRNA (Dallas and Noller, 2001; Lancaster and Noller, 2005; Selmer *et al*., 2006; Fabbretti *et al*., 2007).

There are several reports suggesting that IF3 can also interact with the 70S ribosome·mRNA complex or be present transiently with the 70S IC (Singh *et al*., 2005; Fabbretti *et al*., 2007; Grigoriadou *et al*., 2007). However, the prevailing consensus is that IF3 and 50S subunit binding to the 30S subunit are mutually exclusive (Karimi *et al*., 1999; McCutcheon *et al*., 1999; Dallas and Noller, 2001; Peske *et al*., 2005; Antoun *et al*., 2006; Hirokawa *et al*., 2006), as both IF3 and the 50S subunit appear to bind to the same region of the 16S rRNA. Both the C-terminal domain of IF3 (Tapprich *et al*., 1989; Dallas and Noller, 2001; Fabbretti *et al*., 2007) and helix H69 of the 23S rRNA interact with helices h24 and h45 of the 16S rRNA, interaction of H69 with the 16S rRNA being important for the formation of the B2b intersubunit bridge (Yusupov *et al*., 2001; Ali *et al*., 2006).

Our interpretation that translation re-initiation begins with a 30S ribosome·mRNA complex contrasts with that of others who believe that a 70S ribosome is involved (Petersen *et al*., 1978; Janosi *et al*., 1998; Inokuchi *et al*., 2000; Karamyshev *et al*., 2004; Moll *et al*., 2004). This belief is based partly on experiments showing that inactivation of ribosome-recycling factor (RRF) – required for dissociation of the 70S ribosome following translation termination (Hirokawa *et al*., 2006) – had minimal effects on the efficiency of re-initiation *in vivo* (Janosi *et al*., 1998; Inokuchi *et al*., 2000; Karamyshev *et al*., 2004). Although both the 30S and 70S ribosome may be competent for re-initiation *in vivo*, inactivation of RRF, which leaves the 70S ribosome associated with the mRNA, causes random initiation events from non-canonical start codons, downstream of the translation termination site (Janosi *et al*., 1998; Inokuchi *et al*., 2000). This finding that the ribosome dissociation function of RRF is necessary for accurate re-initiation indicates that a 30S·IF3·mRNA complex and not a 70S·mRNA complex is required for accurate re-initiation *in vivo*. It also differentiates translation re-initiation from translation of leaderless mRNAs, where initiation is thought to involve the 70S ribosome (Moll *et al*., 2004). Re-initiation with a 70S ribosome would additionally require that the ribosome dissociation function of RRF be somehow inhibited at sites specified for translation re-initiation.

## Experimental procedures

Descriptions of plasmid construction and bacterial strains used in this study are in *Supplementary material*, in addition to methods for enzyme assays and tRNA analysis.

## Supplementary material

This material is available as part of the online article from: http://www.blackwell-synergy.com/doi/abs/10.1111/j.1365-2958.2008.06104.x

(This link will take you to the article abstract).
