# Supplementary material for: Requirements for translation re-initiation in Escherichia coli: roles of initiator tRNA and initiation factors IF2 and IF3
Source: Mol Microbiol. 2008 Mar;67(5):1012–26. doi: 10.1111/j.1365-2958.2008.06104.x (PMC2268962; doi:10.1111/j.1365-2958.2008.06104.x)

Supplementary information

Experimental procedures

Bacterial strains and plasmids

E. coli strains used are as follows: **CA274** (HfrH lacZam trpEam), **XL1-Blue** (F'::Tn10 $proA^+$ B^+ $lacI^q$ Δ(lacZ) M15/recA1 endA1 gyrA96 (Nal^r) thi hsdR17(r_k^- m_k^+) glnV44 relA1) and **JM109** (F'TraD36 $proA^+$ B^+ $lacI^q$ Δ(lacZ) M15Δ(lac proAB) recA1 endA1 gyrA96 (Nal^r) thi hsdR17(r_k^- m_k^+) glnV44 relA1 recA1). The pBAD/*myc*-His(B), pQE-60 and pSP-*luc*+ plasmids were obtained from Invitrogen, Qiagen and Promega, respectively. Construction of the pRSVCAT*am1.2.5* and pRSVCAT*2.5* plasmids; pACD plasmid and its derivatives containing genes for IF2, IF3, MetRS and MTF derived from pACYC184 are described elsewhere (Varshney and RajBhandary, 1990; Mangroo and RajBhandary, 1995).

Construction of CAT-fLuc di-cistronic and fLuc mono-cistronic reporters

Using M13 RF DNA as a template, a DNA fragment containing the entire *geneV* and part of *geneVII* was amplified by PCR using the oligonucleotides 5' CGGA*AGATCT*ATGATTAAAGTTGAAATTAAACC 3' as the forward primer and 5' CATG*CCATGG*TGTCGAAATCCGCGACCTGCTCC 3' as the reverse primer (sequences in italics denoting BglII and NcoI restriction sites, respectively). The PCR product was digested with BglII and NcoI, and cloned between the respective sites of the luciferase expression vector pSP-*luc*+. The methionine initiation codon of the firefly

luciferase (fLuc) gene was altered to AUU using site-directed mutagenesis (Quikchange™, Stratagene) and a KpnI site was introduced into *geneV* using the following mutagenic oligonucleotides, 5' CAGTTC*GGTACC*CTTATGATTGAC 3' and 5' GTCAATCATAAG*GGTACC*GAACTG 3'. The resulting construct was digested with KpnI and XbaI to obtain a DNA fragment coding for a section of the C-terminal end of *geneV* and the entire fLuc gene fused downstream to a section of the N-terminal end of *geneVII*. This DNA fragment was ligated to pBAD*myc*-His(B) that was previously digested with KpnI and XbaI. The resulting plasmid containing *geneV-geneVII_*fLu*c* was then digested with NcoI and KpnI. Using pRSVCAT*2.5* as a template, the chloramphenicol acetyltransferase (CAT) gene was amplified by PCR using the following forward and reverse primers, 5' CCGGG*TCATGA*ACAAAAAAACCACTGGATAT 3' and 5' GCCC*GGTACC*CCCGCCCTGCCACTCATCGCAG 3'. The PCR product was digested with BspHI and KpnI, and ligated to the above pBAD*myc*-His(B) based plasmid containing *geneV-geneVII_*fLuc. The resulting plasmid pB-CL (CL) contained the artificial operon encoding CAT*_geneV* and *geneVII_*fLuc fusion proteins, under control of the arabinose promoter.

Mutant and wild type *E. coli* initiator tRNA genes were isolated from plasmids previously described (Mangroo and RajBhandary, 1995), using BamHI and PstI. The DNA inserts contained the tRNA genes under control of their endogenous transcriptional promoters derived from *E. coli* $tRNA_2^{fMet}$. To clone these tRNA genes into CL, a PstI-BglII linker was created and inserted into the BstZ171 site of pBAD*myc*-His(B).

Using the wild type di-cistronic reporter as the template, the mono-cistronic fLuc reporter L was created by PCR amplification of the geneVII_fLuc fusion protein using the following forward and reverse primers, 5' GCCATG*CCATGG*AGCAGGTCGCGGATTTCG 3' and 5' CGTC*TCTAGA*ATTACACGGCGATC 3'. This DNA insert contained a 5' NcoI and 3' XbaI site and was cloned into pBAD-*myc*His(B) plasmid digested with NcoI and XbaI. In this construct, the monocistronic *geneVII_fLuc* gene is under control of the arabinose promoter.

Construction of mutant reporters

Mutant di-cistronic reporters containing UAG or GUC as initiation codons were generated as described below. To generate the mutant reporters $C_{am1}L$ and CL_{am1}, oligonucleotides, 5' GGAATTAACCTAGAACAAAAAAACCAC 3' and 5' CCGGCTAAGTAACTAGGAGCAGGTCGCGG 3' (complementary oligonucleotides not shown) were used respectively, using wild type reporter CL as a template. To create the $C_{GUC1}L$ and CL_{GUC1} mutant reporters, 5' CAGGAGGAATTAACCGTCAACAAAAAAACC 3' and 5' CCGGCTAAGTAACGTCGAGCAGGTCGCGG 3' were used, respectively. The mutant mono-cistronic fLuc reporter initiating with a UAG start codon (L_{am1}) was generated using the mutagenic oligonucleotide 5'GGAATTAACCTAGGAGCAGGTCGCG 3'. Mutant di-cistronic reporters containing UAG stop codons at various positions within the CAT gene were constructed as follows.

Oligonucleotide 5’ GCAGGGCGGGTAGGCCCTTATGATTGACC 3’ was used to generate the mutant di-cistronic reporter C_{am219}L with a 40 nucleotide separation between the CAT and fLuc reporters, while the oligonucleotides 5’ GCCTCGTTCCGTAGAAGTAACATGG 3’ and 5’ CTCGTTCCGGCTTAGTAACATGGAGC 3’ and their respective complements were used to generate mutant di-cistronic reporters with 7 and 4 nucleotide separations, respectively. The mutant di-cistronic reporter with a ribosome binding site (rbs; Shine-Dalgarno sequence) upstream of the fLuc reporter (C_{rbs}L) was created using the mutagenic oligonucleotide 5’ CGTCTGCGCCTCGTTGAGGCTAAGTAACATGGAGC 3’ and its complement. The same mutagenic primers were also used to construct the $C_{amb219/rbs}$L mutant di-cistronic reporter by using C_{amb219}L plasmid as template for mutagenesis.

Induction of reporter system and overexpression of various proteins from the translation machinery

E. coli CA274 transformed with reporter plasmids were grown in LB media containing ampicillin (100 μg/ml). Cells co-transformed with a second expression plasmid containing genes for (IF2, IF3, MetRS or MTF) or the empty vector pACD, were grown in LB media also containing tetracycline (20 μg/ml) and were induced with 0.5 mM IPTG. For induction of the reporter system for short periods of time, single colonies of transformed cells were picked for inoculation in LB media containing the required antibiotics for overnight growth. 25 μl of the overnight cultures were subcultured into 3

ml of fresh media and grown to mid-log phase (0.5-0.8 OD_{600}) before inducing with 0.01 % - 0.1 % arabinose (final concentration) for 30-60 minutes at 37° C. Cultures were placed on ice for 15 minutes and the optical density was measured. Constitutive or long-term induction of the reporter system was performed with the following changes in protocol. Colonies picked from fresh transformation plates were inoculated into LB media containing the required antibiotic(s) and 0.25% arabinose. Cultures were allowed to grow to late log phase (~ 1-1.5 OD_{600}) before being harvested and samples prepared as previously described. Cell extracts prepared from these cells were subsequently analyzed for CAT and fLuc activities. Overproduction of IF2, IF3, MTF and MetRS was verified by immunoblot analysis (IF2, IF3, MTF) or by measuring enzyme activity in crude cell extracts (MetRS).

Assay for luciferase activity

After induction of *E. coli* CA274 transformants, cultures were placed on ice for 15 minutes. 50 μl of the cultures were each mixed with 400 μl of a control culture (*E. coli* CA274 transformed with the empty pBAD/*myc*-His(B) vector) to dilute the samples. 90 μL of the mixed culture were then mixed with 10 μL of 1 M K_2HPO_4 pH 7.8, 20 mM EDTA and quick-frozen on dry ice for at least 10 minutes. The frozen cell suspensions were thawed in a water bath at room temperature and mixed with 300 μL of lysis mix (33 mM K_2HPO_4, 0.67 mM EDTA, 1.7 mg/ml lysozyme, 1.33× cell culture lysis reagent (CCLR, Promega), 3.3 mg/ml BSA) and incubated at room temperature for 10 minutes. Typically, 2 μL of the cell lysate was added to 10 μL of luciferase assay reagent (Promega) and analyzed using a Berthold Sirius luminometer (2 second delay, 10 second

measurement time). fLuc activity was reported as relative luminescence units (RLU), and normalized to cell number, provided by the optical density, for each sample (RLU/OD_{600}). In addition, fLuc was also normalized to CAT activity. Results of the fLuc activity assays are presented as the means and standard deviations from at least 3-5 independent experiments.

Preparation of cell extracts for assays of CAT and β-lactamase activities

Pellets from 1.2 ml of cell culture were re-suspended in 120 μl TME buffer (25 mM Tris-HCl pH 8.0, 2 mM β-mercaptoethanol, 1 mM EDTA) containing 0.1 mg/ml lysozyme and incubated at room temperature for 10 minutes with periodic mixing. 1 μl of DNase I (1U/μl) (New England Biolabs) and $MgCl_2$ to 10 mM were added and incubated for an additional 10 minutes. The digestion mixtures were centrifuged at 13,000 × *g* for 10 minutes at 4° C. 4-8 μl of the supernatant were used for protein determination, while the rest was mixed with an equal volume of 2× protein storage buffer (70 % glycerol, 20 mM Tris-HCl pH 8.0, 10 mM β-mercaptoethanol, 200 mM NaCl) and stored at -20° C.

Assay for CAT activity

Typically, 0.5 μg of total cell extract was incubated in a 100 μl reaction containing 470 mM Tris-HCl pH 8.0, 0.8 mM CoA, 160 μM ^{14}C-chloramphenicol (specific activity of 12.5 μCi/μmole) for 5 minutes at 37° C. Reactions were stopped by addition of ethyl acetate and the upper ethyl acetate layer dried and re-dissolved in 8 μl of ethyl acetate. The reaction products were spotted onto Silica gel thin layer chromatography (TLC)

plates (J.T. Baker, Baker-flex) and separated using a 95 % chloroform/5 % methanol solvent mixture. Autoradiography was performed to visualize spots corresponding to acetylated and non-acetylated chloramphenicol. CAT activity was calculated based on the percentage of acetylated chloramphenicol formed, relative to the total amount of chloramphenicol present. CAT activity was also normalized to β-lactamase activity to normalize for fluctuations in plasmid copy (((acetylated chloramphenicol/total chloramphenicol)/μg protein)/β-lactamase activity). Results of the CAT activity assays are presented as the means and standard deviations from at least 3-5 independent experiments.

Assay for β-lactamase activity

β-lactamase activity was used to normalize *de novo* translation initiation activity to plasmid copy number (i.e. CAT activity from the CL reporter and fLuc activity from the monocistronic L reporter). A stock solution of Nitrocefin (CalBiochem) (500 μg/ml) was diluted ten-fold with 100 mM sodium phosphate pH 7.0 buffer, 1 mM EDTA. 1 μg of total cell extract was added to 1 ml of diluted nitrocefin solution and incubated at room temperature for 5-10 minutes. The reaction was stopped by the addition of 110 μl of 10 % SDS and the absorbance measured at 486 nm using nitrocefin solution without added enzyme as a blank.

SDS-polyacrylamide gel electrophoresis (PAGE) and immunoblot analysis

Cell pellets were suspended in 1×SDS-PAGE sample buffer (62.5 mM Tris-HCl pH 6.8, 2 % SDS (w/v), 10 % β-mercaptoethanol (v/v), 10 % glycerol (w/v) and 0.01 % bromophenol blue). Typically, protein from 2.5×10^7 cells were analyzed by PAGE and subsequently blotted onto the transfer membrane (Immobilon-P (Millipore)). Membranes were blocked with blocking buffer (Tris buffered saline (TBS), 20 mM Tris-HCl pH 7.5, 140 mM NaCl + 5 % milk powder) and washed with TBS + 0.05 % Tween-20. Incubation of the membrane with primary antibody or with secondary horse radish peroxidase (HRP)-coupled secondary antibody was in blocking buffer for 60 minutes at room temperature. The blots were developed using Chemiluminescent (ECL) reagent (Pierce) and exposed to X-ray film (Kodak XAR).

Densitometric quantitation of signals from immunoblots

Protein expression levels were quantitated by densitometric analysis of immunoblots using the FluorChem® HD2 Imaging system (Alpha Innotech Corp.). Cell extracts from *E. coli* expressing mutant CAT protein and extracts from cells expressing wild type CAT were analyzed using immunoblots. A series of dilutions of cell extract from the wild type CAT sample was analyzed and quantitated to generate standard curves to extrapolate the relative amount of mutant CAT protein expressed.

Isolation of total tRNA from E. coli under acidic conditions

An overnight culture of the desired *E. coli* strain was used to inoculate 25 ml of LB media and grown at 37° C until a OD_{600} ~ 1.2–1.5 was reached. Cultures were chilled on

ice and centrifuged at 5,000 *g* for 10 minutes. Cells were harvested and the pellet re-suspended in 200 μl of 0.1 M sodium acetate pH 5. Trizol reagent (Invitrogen) was then used following the manufacturer's instruction to isolate total RNA under acidic conditions. The resulting RNA pellet was dried on ice, dissolved in 100 μl of 10 mM sodium acetate pH 5.0, 1 mM EDTA and quick frozen and stored at –80° C. RNA concentration was determined by measuring the A_{260}. Base catalyzed deacylation of aminoacyl tRNA was performed, when necessary, by incubating samples in 0.2 M Tris-HCl pH 9.0 at 37° C for 30 minutes.

Acid urea-PAGE/Northern blot analysis

0.075 A_{260} of total tRNA was mixed with 1 volume of acid urea sample buffer (100 mM sodium acetate pH 5.0, 7 M urea, 0.5 % bromophenol blue, 0.5 % xylene cyanol). The samples were then subjected to electrophoresis on a 6.5 % polyacrylamide (19:1) gel containing 100 mM sodium acetate buffer pH 5.0, 8 M urea at 500 V for ~ 14 hours at 4° C as described by Varshney *et al* (Varshney *et al.*, 1991). The portion of the gel containing tRNA was transferred to Hy-Bond N^{+} blotting membrane (Amersham Biosciences). Transfer was performed in 10 mM Tris-acetate pH 7.8, 5 mM sodium acetate, 0.5 mM EDTA at 10 V for 20 minutes and 40 V for two hours. The blot was per-hybridized in 6×SSC / 0.5 % SDS / 10× Denhardt's buffer for 3 hours at 42° C and hybridized with a 5' ^{32}P-labelled DNA oligonucleotide probe in 6×SSC / 0.5 % SDS for 12 hours at 42° C. The following DNA oligonucleotides were used to identify expression of the mutant initiator tRNAs: 5' TGGCCGCGGGGGCCGGATT 3', G72G73/U35A36 mutant initiator tRNA (G72G73); 5' CGACGATCTTCGCGTTTAGA 3',

C30:G40/U35A36 mutant initiator tRNA (C30G40); and 5’ CGACGATCTTCTCATTTAGA 3’, U29C30A31:U39G40U41/U35A36 mutant initiator tRNA (3GC). The blots were washed three times with 6×SSC / 0.1 % SDS at room temperature before exposure to film.

M13 Bacteriophage experiments

Bacteriophage experiments were performed as described (Sambrook and Russell, 2001). Plating bacteria were prepared by streaking *E. coli* cultures or transformants onto LB agar plates, and single colonies picked for inoculation into LB liquid media. Cultures were then grown at 37° C until OD_{600} of ~ 0.2 was reached, placed on ice and stored at 4° C. Bacteriophage M13 was plated by incubating 100 μl of plating bacteria with a desired amount of bacteriophage stock and mixed with molten LB top agar (0.75 %) containing 5 mM $MgCl_2$ pre-incubated at 47° C. The infection mixture was then poured onto LB agar (1.5 %) plates containing 5 mM $MgCl_2$. Once the agar solidified, the plates were inverted and incubated at 37° C typically for 8-12 hours. When necessary, X-Gal and IPTG were added to the LB top agar before plating. To determine phage titer, 100 μl of plating bacteria was subcultured into 3 ml of LB media containing 5 mM $MgCl_2$ and grown at 37° C with shaking for 2 hours. 10-100 μl of diluted bacteriophage suspension was then added to the liquid cultures and the infected cultures were incubated with shaking for a further 6 hours at 37° C or as needed. Cultures were centrifuged at 13,000 × *g* for 5 minutes. The supernatants containing soluble infectious M13 bacteriophage were then stored at 4° C or diluted appropriately to infect either JM109 or CA274 cells to determine phage titer. When utilizing transformants, the appropriate antibiotics were also added.

REFERENCES

Mangroo, D., and RajBhandary, U.L. (1995) Mutants of Escherichia coli initiator tRNA defective in initiation. Effects of overproduction of methionyl-tRNA transformylase and the initiation factors IF2 and IF3. *J Biol Chem* **270**: 12203-12209.

Sambrook, J., and Russell, D.W. (2001) *Molecular Cloning: A Laboratory Manual.* Cold Spring Harbor, New York, USA: Cold Spring Harbor Laboratory Press.

Varshney, U., and RajBhandary, U.L. (1990) Initiation of protein synthesis from a termination codon. *Proc Natl Acad Sci U S A* **87**: 1586-1590.

Varshney, U., Lee, C.P., and RajBhandary, U.L. (1991) Direct analysis of aminoacylation levels of tRNAs in vivo. Application to studying recognition of Escherichia coli initiator tRNA mutants by glutaminyl-tRNA synthetase. *J Biol Chem* **266**: 24712-24718.

Supplemental Figure legends

S1. Di-cistronic expression vector pBAD derived plasmid used to express the CAT and fLuc fusion reporters using an arabinose-inducible promoter. Wild type or mutant initiator tRNA genes were also expressed from the same plasmid.

S2. *De novo* initiation of mono-cistronic fLuc reporter using a UAG initiation codon **(Top)** fLuc activity from *E. coli* CA274 transformed with mono-cistronic fLuc reporters initiating with a AUG (L, white bars) or a UAG (L_{am1}, grey bars) start codon and co-expressing the wild type initiator tRNA (fMet) or the U35A36 mutant initiator tRNA. fLuc activity is defined as (relative luminescence units (RLU)/cell number)/β-lactamase activity. **(Bottom)** Immunoblot of cell extracts from the transformants above, using anti-fLuc antibody.

S3. Mutant initiator tRNAs defective in formylation of aminoacyl-tRNA or in ribosomal P-site binding. **(A)** Structure of the U35A36 mutant initiator tRNA containing additional mutations in the acceptor stem (G72G73) or in the anticodon stem C30:G40(C30G40), U29C30A31:U39G40A41(3GC). **(B)** Acid urea polyacrylamide gel electrophoresis/Northern blot analysis of total tRNA isolated under acidic conditions from *E. coli* CA274 transformed with di-cistronic reporters containing specified mutant initiator tRNA genes.

S4. Overexpression of IF3 reduces re-initiation efficiency *E. coli* CA274 cells were co-transformed with the CL reporter and expression plasmids that were either empty (ACD, grey bar), or contained genes encoding IF2, IF3, MetRS or MTF (white bars). Transformants were induced with arabinose and cell extracts analyzed for CAT activity, normalized to β-lactamase activity **(A)** and fLuc activity normalized to CAT activity **(B)**.

S5. Overexpression of IF3 does not significantly affect *de novo* translation of the fLuc reporter *E. coli* CA274 cells were co-transformed with the mono-cistronic L reporter and an expression plasmid that was either empty (ACD, grey bar) or contained the IF3 gene (white bar). fLuc activity was normalized to β-lactamase activity and the activity from cells not overproducing IF3 (ACD) set at 100 %.

Table S1. Translational efficiency of the U35A36 mutant initiator tRNA in *de novo* initiation of CAT and re-initiation of fLuc determined using immunoblots and densitometric analysis.

Reporter	% activity relative to wild type reporter
CL	100
$C_{am1}L$	17 ± 1.5
CL_{am1}	6.6 ± 0.3

Values presented as mean ± S.D.

Fig. S1

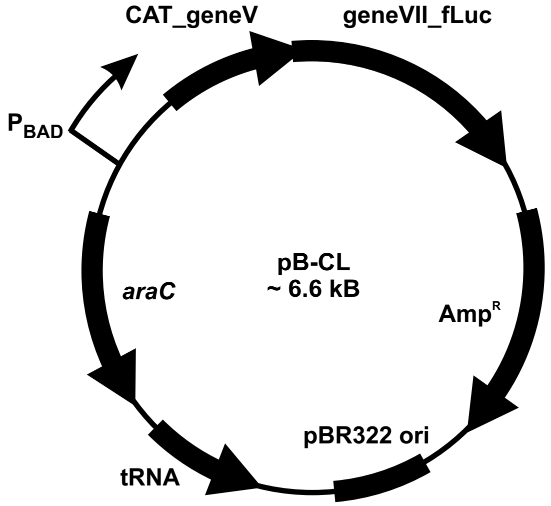
CAT_geneV
geneVII_fLuc
P_{BAD}
araC
pB-CL
~ 6.6 kB
Amp^R
pBR322 ori
tRNA

Fig. S2

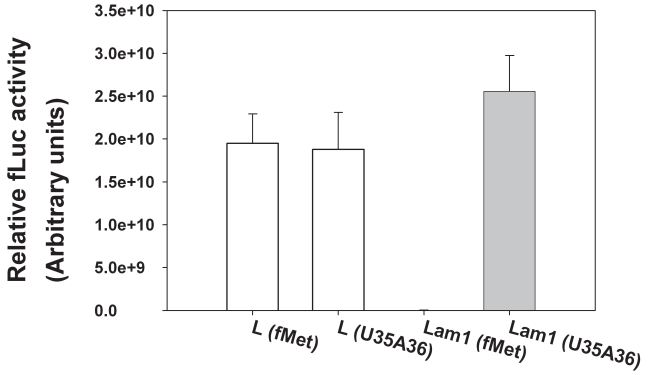

lane	1	2	3	4	5
fLuc start codon	-	AUG	AUG	UAG	UAG
tRNA	-	fMet	U35 A36	fMet	U35 A36

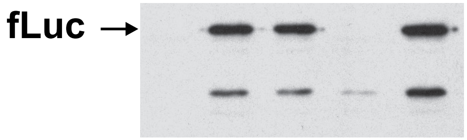

Fig. S3

A

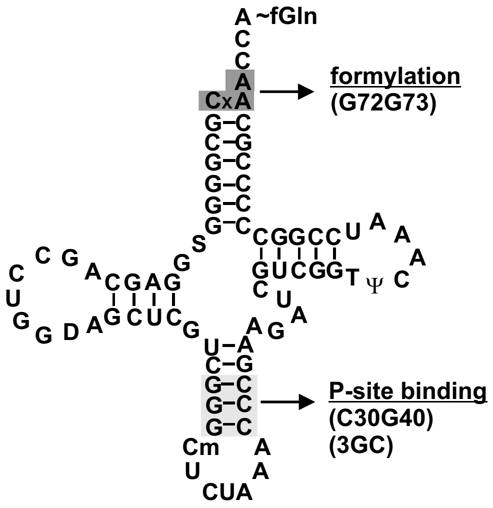

B

lane	1	2	3	4
tRNA	G72G73	G72G73	C30G40	C30G40
OH^-	-	+	-	+

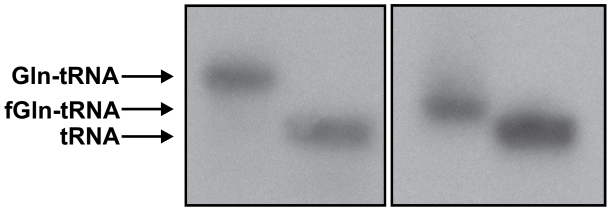

Fig. S4

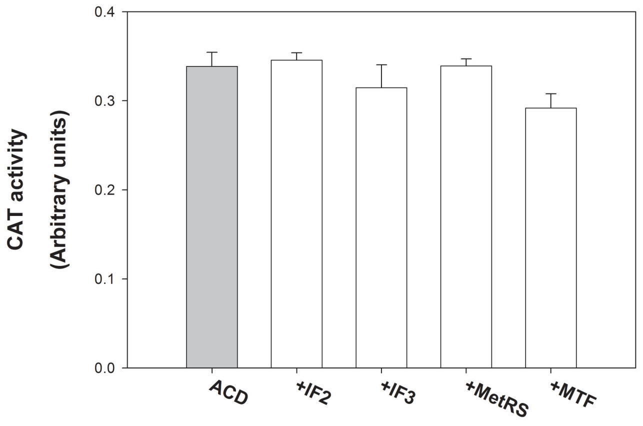

CAT activity
(Arbitrary units)
0.4
0.3
0.2
0.1
0.0
ACD
+IF2
+IF3
+MetRS
+MTF

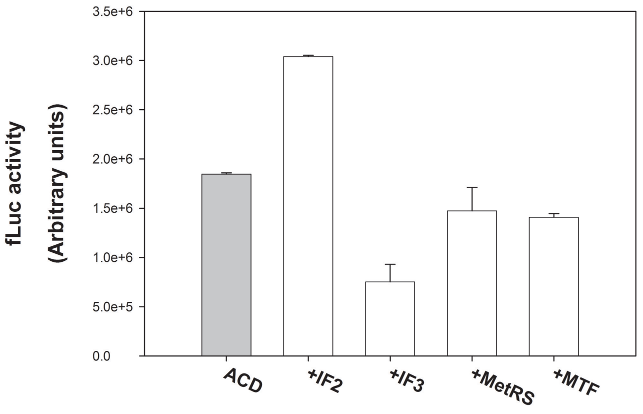

fLuc activity
(Arbitrary units)
3.5e+6
3.0e+6
2.5e+6
2.0e+6
1.5e+6
1.0e+6
5.0e+5
0.0
ACD
+IF2
+IF3
+MetRS
+MTF

Fig. S5

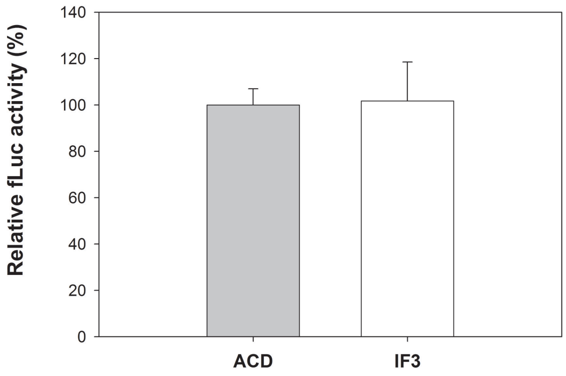

Supplement: Supplementary file 1 [file mmi0067-1012-SD1.pdf]
